# Tyree's Antiseptic Powder

**Published:** 1904-11

**Authors:** 


### TYREE'S ANTISEPTIC POWDER

CONTAINS

Sod. bor., alumen, ac. carbol., glycerin, the cryst. principles of thyme, eucalyptus, gaultheria and mentha.

For Ulcerated conditions of the mucous membrane, one or two teaspoonfuls to a pint of water three or four times a day. For Scrofulous, Syphilitic and Varicose Ulcers, apply the powder full strength or dilute with Boracic Acid. As an ointment, use from one to three drachms to one ounce of petroleum. For spraying the nose and throat, from twenty-five to one hundred grains to one pint of water (dissolves immediately). For *immediate* deodorizing and disinfecting, sprinkle the Powder direct upon the object affected; the results will be instantaneous. For Prickly Heat, Poison Oak, Squamous Eczema and other conditions of a similar nature, use from one to eight teaspoonfuls to a pint of water (has proven very serviceable for these conditions). As a deodorant and prophylactic in dental work, use one or two teaspoonfuls to a pint of water. For disinfecting offensive cavities, fill them with the Powder. For profuse and offensive perspiration, swelling, soreness and burning of the body and feet, use full strength or diluted with water. As a delightful toilet preparation after the bath and shaving, from one to two teaspoonfuls to a pint of water. The price is practically nothing. Ten cents worth will make one gallon of standard solution.

